# Supplementary material for: From sequence to enzyme mechanism using multi-label machine learning
Source: BMC Bioinformatics. 2014 May 19;15:150. doi: 10.1186/1471-2105-15-150 (PMC4229970; doi:10.1186/1471-2105-15-150)
Supplement: Additional file 2 — Java code of ml2db. Additional file ml2db_code.tar.gz contains the Java source code to run the multi-label machine learning experiments and save the results to database. The code’s Javadoc is included. [file 1471-2105-15-150-S2.zip › additional file 2/ml2db/utils/doc/uk/ac/standrews/utils/main/PropertiesUtils.html]

PropertiesUtils


JavaScript is disabled on your browser.


- Overview
- Package
- Class
- Use
- Tree
- Deprecated
- Index
- Help

- Prev Class
- Next Class

- Frames
- No Frames

- All Classes

- Summary:
- Nested |
- Field |
- Constr |
- Method

- Detail:
- Field |
- Constr |
- Method


uk.ac.standrews.utils.main

## Class PropertiesUtils

- java.lang.Object
- - uk.ac.standrews.utils.main.PropertiesUtils

- ---

    

  ```
  public class PropertiesUtils
  extends java.lang.Object
  ```

  Utilities to manipulate Java properties and properties text files

  Version:
  :   4 Jan 2008

  Author:
  :   Luna De Ferrari luna.deferrari-at-ed.ac.uk

- - ### Constructor Summary

    Constructors

    Constructor and Description

    `PropertiesUtils()`
  - ### Method Summary

    Methods

    | Modifier and Type | Method and Description |
    | --- | --- |
    | `static void` | `getJavaSystemProperties()` Gets the Java system properties |
    | `static java.util.Vector<java.lang.String>` | `getKeysStrings(java.util.Properties props)` Get the properties keys as strings |
    | `static java.util.Vector<java.lang.String>` | `getValuesStrings(java.util.Properties props)` Get the properties values as strings |
    | `static java.util.Properties` | `readLocalProperties(java.lang.String resourceName)` Reads properties that inherit from three locations. |
    | `static java.util.Properties` | `readProperties(java.lang.String propertiesFilePath)` Reads properties Code adapted from Weka: http://www.cs.waikato.ac.nz/ml/weka/ Ian H. |

    - ### Methods inherited from class java.lang.Object

      `equals, getClass, hashCode, notify, notifyAll, toString, wait, wait, wait`

- - ### Constructor Detail


    - #### PropertiesUtils

      ```
      public PropertiesUtils()
      ```
  - ### Method Detail


    - #### getJavaSystemProperties

      ```
      public static void getJavaSystemProperties()
      ```

      Gets the Java system properties


    - #### getKeysStrings

      ```
      public static java.util.Vector<java.lang.String> getKeysStrings(java.util.Properties props)
      ```

      Get the properties keys as strings

      Parameters:
      :   `props` - a properties object

      Returns:
      :   a vector with one string for each key in the properties


    - #### getValuesStrings

      ```
      public static java.util.Vector<java.lang.String> getValuesStrings(java.util.Properties props)
      ```

      Get the properties values as strings

      Parameters:
      :   `props` - a properties object

      Returns:
      :   a vector with the properties values as strings


    - #### readLocalProperties

      ```
      public static java.util.Properties readLocalProperties(java.lang.String resourceName)
                                                      throws java.lang.Exception
      ```

      Reads properties that inherit from three locations. Code from Weka:
      http://www.cs.waikato.ac.nz/ml/weka/
      Ian H. Witten and Eibe Frank (2005)
      "Data Mining: Practical machine learning tools and techniques", 2nd
      Edition, Morgan Kaufmann, San Francisco, 2005.
      Reads properties that inherit from three locations. Properties are first
      defined in the system resource location (i.e. in the CLASSPATH). These
      default properties must exist. Properties defined in the users home
      directory (optional) override default settings. Properties defined in the
      current directory (optional) override all these settings.

      Parameters:
      :   `resourceName` - the location of the resource that should be loaded. e.g.:
          "weka/core/Utils.props". (The use of hardcoded forward slashes
          here is OK - see jdk1.1/docs/guide/misc/resources.html) This
          routine will also look for the file (in this case)
          "Utils.props" in the users home directory and the current
          directory.

      Returns:
      :   the Properties

      Throws:
      :   `java.lang.Exception` - if no default properties are defined, or if an error
          occurs reading the properties files.


    - #### readProperties

      ```
      public static java.util.Properties readProperties(java.lang.String propertiesFilePath)
                                                 throws java.lang.Exception
      ```

      Reads properties Code adapted from Weka:
      http://www.cs.waikato.ac.nz/ml/weka/
      Ian H. Witten and Eibe Frank (2005)
      "Data Mining: Practical machine learning tools and techniques", 2nd
      Edition, Morgan Kaufmann, San Francisco, 2005.

      Returns:
      :   the Properties

      Throws:
      :   `java.lang.Exception` - if no default properties are defined, or if an error
          occurs reading the properties files.


- Overview
- Package
- Class
- Use
- Tree
- Deprecated
- Index
- Help

- Prev Class
- Next Class

- Frames
- No Frames

- All Classes

- Summary:
- Nested |
- Field |
- Constr |
- Method

- Detail:
- Field |
- Constr |
- Method
